# Optimal Management of Spontaneous Aortic Thrombus Floating in the Ascending Aorta, from a Single Case Experience to a Literature Review

**DOI:** 10.3390/jcdd12040146

**Published:** 2025-04-09

**Authors:** Jacopo Gardellini, Daniele Linardi, Venanzio Di Nicola, Gino Puntel, Giovanni Puppini, Luca Barozzi, Giovanni Battista Luciani

**Affiliations:** 1 Cardiac Surgery Department, Azienda Ospedaliera Universitaria Integrata Verona, 37126 Verona, Italy; daniele.linardi@aovr.veneto.it (D.L.);; 2 Radiology Department, Azienda Ospedaliera Universitaria Integrata Verona, 37126 Verona, Italy

**Keywords:** ascending aorta, thrombus, ulcerated plaque, spontaneous thrombosis

## Abstract

As spontaneous ascending aortic thrombi (AATs) are uncommon in modern clinical practice, despite the application of new technology and the widespread use of contrast-enhanced computer tomography during primary assessments in patients without underlying predisposing conditions, a thrombus floating in the ascending aorta is rarely discovered in a timely manner; moreover, the ascending tract represents an unusual site for thrombus formation. The clinical presentation of AATs is also often in the form of peripheral arterial embolization, which can cause a wide variety of symptoms, from stroke to limb ischemia, and thus delay correct diagnosis. Medical management is a risky strategy, while surgical treatment is usually challenging due to the risk of thrombus dislodgement and difficulties related to prior embolization complication management. In this study, faced with a peculiar case of embolic stroke in an otherwise healthy 71-year-old woman, we analyzed the status of knowledge on spontaneous ascending aortic thrombus treatments and outcomes. A multidisciplinary approach represents the best choice for defining a patient’s timing of surgery and ensuring the management of complications. Sequential multistage treatment minimizes further complications and prevents worsening patient outcomes, leading to the best management for every possible clinical presentation. A less invasive surgical approach could lead to complete resolution of the pathology, avoiding further potentially lethal complications, facilitating postoperative management, avoiding delayed treatments, and resulting in better outcomes.

## 1. Introduction

Ascending aortic thrombi (AATs) are rare findings usually caused by preexisting aortic conditions such as atherosclerosis or acute events such as aortic dissection (AD) or traumatic aortic injuries (TAIs). The incidence rate for an aortic mural thrombus without a definite underlying cause, which can lead to AATs, is about 0.45% [1].

Both structural pathologies and medical conditions, such as hypercoagulability, tobacco use, malignancies, medication use, autoimmune diseases, acute or chronic inflammation, primary endothelial disorders, and vasculitis, could promote clotting, thus leading to aortic thrombus formation [2]. Additionally, aortic thrombi are commonly detected in the thoracic descending aorta or the aortic arch, making the ascending aorta the most uncommon location for thrombi to develop due to its high-blood-flow environment and sheer wall stress [3].

Currently, no unique treatment exists for this pathology. However, the fact that embolization could lead to catastrophic effects underlines the importance of a rapid, punctual treatment strategy that could prevent further complications and solve the acute problem.

## 2. Case Presentation

**Figure 1:**
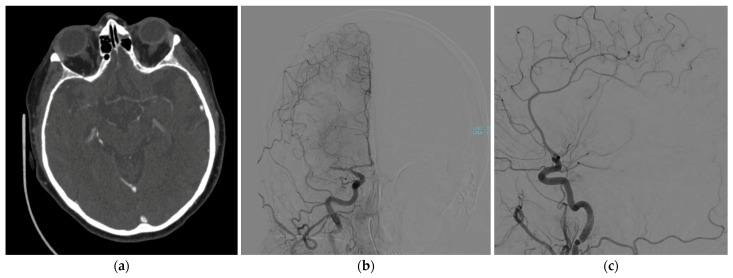
Brain CTA showing a complete occlusion of the right middle cerebral artery in (**a**) axial view; confirmed during a cerebral angiography: (**b**) coronal and (**c**) sagittal views.

A 71-year-old woman was admitted to our hospital with sudden left hemisyndrome and dysarthria. The patient was affected by empty sella syndrome and bipolar disorder under medication and had a recent hospital admission for dyspnea, where her chest computed tomography angiography (CTA) was negative for pulmonary embolism or pneumonia. Her nucleic acid test for COVID-19 was also negative. After further neurological evaluation, clinical suspicion of a stroke was confirmed, also presenting neglect and anosognosia, with a National Institutes of Health Stroke Scale score of 10. An urgent CTA was performed, showing complete occlusion of the right middle cerebral artery, which caused a massive ischemic stroke (Figure 1).

**Figure 2:**
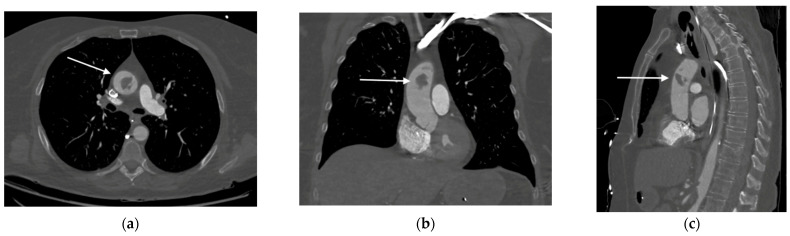
Contrast-enhanced chest CT revealing the presence of a mass (as indicated by the arrows) in the ascending aorta: (**a**) axial; (**b**) coronal; and (**c**) sagittal views.

Due to a suspicion of aortic dissection, a subsequent chest contrast-enhanced CTA was performed, revealing the presence of a 20 mm thrombus in the ascending aorta, 4 cm above the aortic valve and close to the common origin of the brachiocephalic artery and the left common carotid artery, without evidence of atherosclerosis or intimal tear (Figure 2).

**Figure 3:**
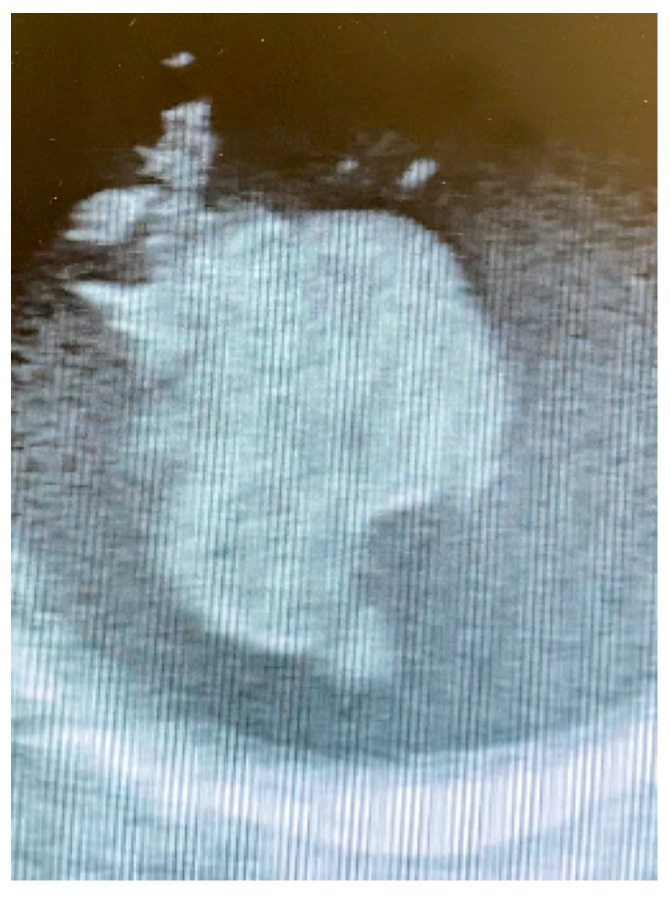
Intraoperative epiaortic echography showing the floating thrombus.

Due to these clinical conditions, a multistage approach was planned after a multidisciplinary evaluation. Prompt mechanical thrombectomy for the MCA occlusion was performed, with optimal results at the following CT scan. The next day, after complete neurological recovery with no residual impairment, the patient underwent a surgical thrombectomy via standard median sternotomy. The femoral artery and right atrium were the selected sites of cannulation; an epiaortic echography confirmed the presence of a floating mass close to the origin of the brachiocephalic artery (Figure 3).

**Figure 4:**
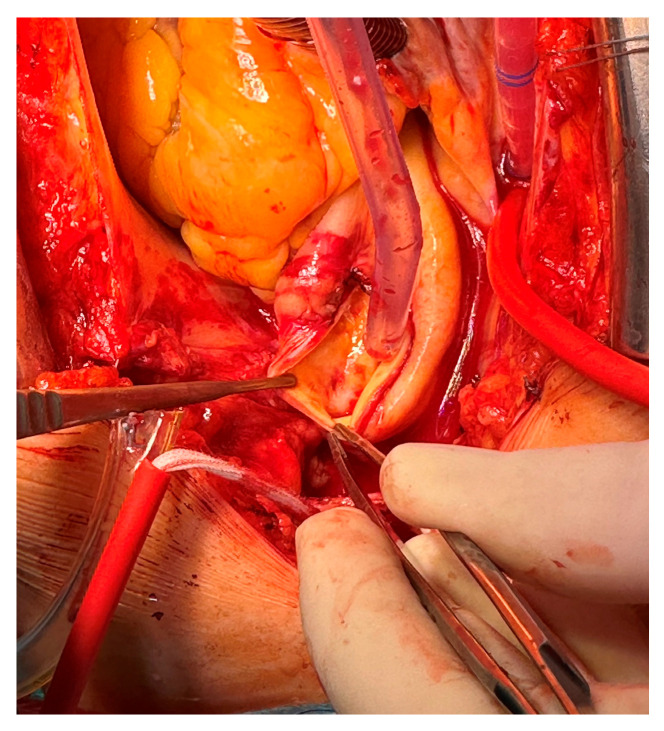
Minimal ulcerated plaque; thrombus attachment site to the aortic wall.

Due to the high risk of embolization connected to the aortic cross-clamp, the surgical strategy was to remove the mass during moderate hypothermic circulatory arrest at 26 °C through a longitudinal aortotomy. The 28 mm × 24 mm thrombus was linked to the aortic wall through a thin peduncle; the attachment site was in the ascending aorta, leading to the mass floating just in front of the innominate artery origin. After a meticulous inspection, a minimal ulcerated aortic plaque was found in the aortic wall (Figure 4).

**Figure 5:**
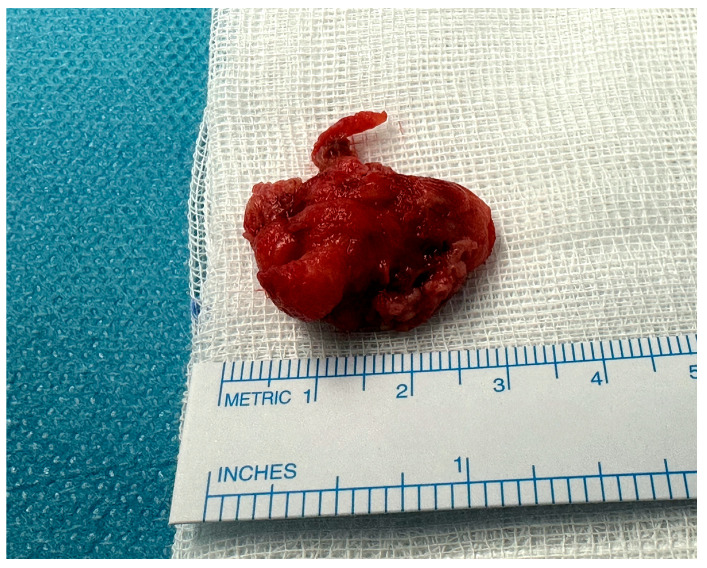
Excised aortic floating thrombus.

To minimize the circulatory arrest time and the risk of further brain ischemic insult, the thrombus was rapidly excised and the aortotomy was closed without ascending aorta replacement. The cardiopulmonary bypass was promptly restarted, with only 8 min of complete hypothermic circulatory arrest. Both the cerebral embolus and aortic mass turned out to be thrombotic formations according to a histological analysis, with classical Zahn lines found in the aortic sample (Figure 5).

A further investigation into genetic coagulation disorders turned out to be negative, with no mutations such as in Leiden V factor, or altered prothrombin; no alterations in APC resistance; and heterozygosity for an A1298C mutation on methylenetetrahydrofolate reductase (MTHFR), with no C677T mutation. Antiphospholipid antibody syndrome (APS) was also determined to be negative, with just a slight alteration in anti-cardiolipin IgM (64.8 U/mL) and normal IgG (15.8 U/mL). Neither malignancies nor infectious diseases were detected during further investigations. After 14 days, the patient was discharged home, with complete neurological recovery, and administered anticoagulation therapy with warfarin after a hematological evaluation. A postoperative CTA confirmed a standard aortic wall; no recurrences were found on a 2-month follow-up CTA scan.

## 3. Methods

The MEDLINE/PubMed database was searched for publications about ascending aortic floating thrombi, including surgical treatment and anticoagulation strategy (Table 1). We restricted the case report research to the last 5 years due to the recent evolution of surgical approaches and new technological developments that have changed aortic pathology treatment. We managed to include all articles with the following keywords: aortic, ascending, thrombus, embolization, and anticoagulation. A manual search of all relevant publications supplemented the article collection. We have excluded publications about severe atherosclerotic aorta, aortic root pathology, and thrombi caused by a well-determined preexisting prothrombotic disease.

## 4. Discussion

Although a rare condition, floating aortic thrombi are associated with early complications such as peripheral embolization and stroke, making its occurrence a potentially life-threatening situation. Typically, its clinical presentation is linked to embolism, with stroke and myocardial infarction being the most common initial signs of aortic thrombus [41].

Effective management of these early complications is crucial as the right approach can help minimize adverse outcomes. In cases of peripheral embolism, while cardiac sources are the most frequent causes, it is important to investigate aortic thrombus even in the absence of any underlying coagulation disorders or structural issues. A floating aortic thrombus is recognized as the source of arterial embolism in 5% to 9% of patients [42,43].

The etiopathogenesis of aortic thrombi is heterogeneous and still not fully understood, although the recent literature has depicted some predisposing factors. Endothelial lesions represent the most common sub-stratum for thrombosis; additionally, in mild atherosclerotic aortas, wall microlesioned plaques are usually the attachment site of a floating mobile mass due to the subsequent deposition of adherent debris [20,44]. Clinical conditions, including inflammation and hypercoagulation, whether genetically determined or acquired, can promote thrombotic manifestations. Notably, the recent COVID-19 pandemic has led to a significant increase in thromboembolic events involving the ascending aorta. This phenomenon is linked to the hyperinflammatory process associated with a hyper-coagulable state as observed in several case reports [45,46]. Smoking habits, including the use of electronic cigarettes, are strongly associated with the formation of blood clots, likely due to the inflammatory response they induce [2,30]. Additionally, the use of certain medications and drugs, such as cocaine, may pose significant risk factors. Thrombotic promotion has also been shown to occur with the use of steroids or chemotherapy agents. Cisplatin, for example, causes endothelial damage and is related to vascular events in about 45% of patients; it is also linked to thrombus formation in the ascending aorta [47,48,49,50,51].

Malignancies pose a risk for systemic thrombosis, including the development of primary aortic tumors. Among these tumors, angiosarcoma is the most common histotype and is closely associated with the formation of aortic thrombus in the ascending aorta [52]. When a primary wall neoplasm is suspected, aortic resection is often necessary to prevent cancer recurrence, and a biopsy is essential for making a definitive diagnosis [11,47].

During a patient’s first assessment, an extensive investigation of possible hypercoagulability and autoimmune disorders should always be conducted. A systemic prothrombotic state could be determined based on numerous medical conditions, including antiphospholipid syndrome, lupus anticoagulant, hyperhomocysteinemia, and endocrine disorders such as hyperprolactinemia [13,22,53].

Structural abnormalities, such as a bovine arch or abnormalities in the supra-aortic vessels, may contribute to the development of a unique flow pattern in the ascending aorta. This altered flow can potentially lead to the formation of thrombi. However, to date, this phenomenon has only been documented in a limited number of cases and requires further research for validation [5,54,55].

As a rare clinical finding, only a few AATs are described in the literature, and still no consensus exists about the best treatment strategy (Table 2). Guidelines about aortic pathology describe the possibility of medical management and surgical treatment of the condition, but a case-by-case evaluation is mandatory to determine the best choice between the two [56].

The decision between a conservative and a surgical approach should depend on the characteristics of the thrombus, with the surgical option being preferred in the presence of significant embolic risk. Thrombus characterization primarily involves assessing its location, with the ascending aorta being the least common site, accounting for only 12% of cases as indicated in a meta-analysis by Fayad and colleagues [57].

The classification proposed by Verma et al. divides the pathological findings by location. Type I includes the ascending aorta up to the origin of the left subclavian artery, including the most dangerous type of thrombi, which can lead to cerebral embolism [58]. This classification could be used as a reference to determine the best treatment: usually, a surgical approach is preferred for type I thrombi to prevent potentially lethal cerebral and cardiac embolism. A previous study has demonstrated that ascending aorta location and prior cerebral embolization are two stronger predictors of embolization recurrence [57].

Morphological features are also important when deciding on AAT management. Based on their relationship with the aortic wall and vessel lumen, aortic thrombi can be classified as sessile, pedunculated, or occlusive [59]. Smaller attachment sites, such as in pedunculated thrombi, have been proven to be linked to severe peripheral embolism compared with mural thrombi (73% vs. 12%) [60]. Yang and colleagues proposed a new index called the break-off risk ratio (boRR), which is calculated as the ratio of the length of the floating portion of a lesion to the length of its attachment point. This index aims to highlight the significance of thrombus morphology and hemodynamics [61]. However, it remains largely theoretical, and further studies are needed to demonstrate its effectiveness. Other researchers have emphasized the importance of evaluating hemodynamics in an aortic floating thrombus to predict the risk of embolization. They encourage the use of advanced imaging techniques, such as cardiac computed tomography, four-dimensional computed tomography angiography (4DTCA), or intra-vascular ultrasound (IVUS), to study floating masses more comprehensively [6,24]. A significant challenge in this area is that clinical presentations are typically acute and often related to embolic complications. As a result, patients are frequently assessed using CTA along with transthoracic and transesophageal echocardiography; additional investigations are usually limited to rare anecdotal instances. Furthermore, monitoring for thrombus via transesophageal echocardiography or epiaortic ultrasonography is recommended during surgical preparation for cardiopulmonary bypass and cannulation to detect any potential adverse embolisms [12,62,63]. Magnetic resonance imaging has rarely been reported as a useful tool in excluding malignancy in particularly challenging cases [64].

Multiparameter evaluation and risk stratification should guide clinicians in choosing the best treatment option between conservative and operative management. Medical therapy, consisting of administering anticoagulant or antiplatelet drugs, is still widely used, especially when surgical risk is prohibitive or embolic risk is low [14,65,66]. In a systematic review by Fayad et al., the mortality rate was similar between conservative and surgical management. However, the complication rate, the recurrence of thrombus formation, and embolic episodes were considerably more frequent after medical treatment alone [57]. A recent meta-analysis described thrombus persistence in approximately one-third of patients after a primarily pharmacological treatment, with a 21% rate of recurrent embolization [41]. Other studies have shown that even one out of five presented the need for a subsequent surgical operation for illness recurrence [67]. A probable explanation could be that usually aortic thrombi are mostly sub-acute or chronic by the time they are detected, making thrombolysis ineffective [17]. In contrast, anti-coagulation treatment appears to be effective for thrombi in the descending aorta and aortic arch, with complete dissolution achieved in 71.4% to 100% of cases [68].

When considering surgical options, choosing a less invasive method whenever possible is best. In some cases, simply removing the thrombus may be sufficient to treat the acute condition without leading to recurrence. Avoiding unnecessary aortic substitution with a prosthetic graft, particularly in cases involving a non-atherosclerotic or lesioned aorta, can help prevent complications related to surgery. Graft replacement involves longer surgery times, and proximal and distal anastomotic lines could lead to endothelial injury. This injury is one of the three factors in Virchow’s triad that promote thrombogenesis, thereby increasing the risk of thrombus recurrence [69].

Recent advancements in technology have expanded the options available for operative approaches. Endovascular and interventional techniques, which are commonly used for treating descending aortic issues, are now being applied, albeit less frequently, to cases involving thrombi in the ascending aorta. Several articles, including a literature review by Meyermann and colleagues, have highlighted the effectiveness of the endovascular approach in excluding thrombi from the descending aorta [70]. However, still, only a limited number of documented cases utilize endovascular stent-grafting or hybrid procedures to treat AATs. Notably, the percutaneous approach using the An-gioVac system has also proven to be feasible for these cases [71,72,73,74]. Furthermore, endovascular surgery procedures have increased remarkably within the last 20 years, to the point that in addition to aortic thrombi, we may face a new problem of in-stent thrombi formation [61,75].

Postoperative management plays a fundamental role in determining patients’ long-term outcomes. Anticoagulation should be used preoperatively and continued after an eventual operation. However, clear evidence on what specific anticoagulant to use remains lacking. Preoperative treatment could vary from warfarin to direct oral anticoagulants. Moreover, regarding secondary prevention, the use of dual antiplatelet therapy or moderate-intensity anticoagulation with warfarin seems to lower the risk of recurrent stroke. In addition, retrospective studies have suggested that anticoagulation is beneficial in patients with mobile mural thrombi [76]. Specific medical conditions could benefit from reasoned medical therapy; for example, prophylactic anticoagulation during cancer chemotherapy may help prevent thromboembolic complications [50].

A regular CTA or at least echocardiographic follow-up is mandatory. This allows for monitoring potential recurrences and designing further treatments to prevent subsequent complications.

## 5. Conclusions

Rare and often spontaneous, aortic thrombosis has a poor prognosis if not identified and treated promptly. Currently, no established guidelines exist for its management, and each treatment option has its advantages and disadvantages. Effective management begins with a thorough preoperative assessment and strategic surgical planning. Thanks to advancements in technology, we can now approach aortic pathologies from innovative perspectives. Catheter-directed thrombolysis can help alleviate the thrombosis to some extent; however, an open aortic thrombectomy is often required. Embracing minimally invasive techniques can significantly lower the risk of complications. Additional research is, therefore, needed to develop recommendations for the best treatment approaches and future monitoring of aortic thrombosis. Postoperative care must not be overlooked; implementing a comprehensive follow-up strategy that includes tailored medical therapy and consistent monitoring is crucial for preventing future embolism and disease recurrences. Prioritizing these steps can lead to improved patient outcomes and a better quality of life.

## Figures and Tables

**Table 1:** Literature review.

Author	Year	Patient Data	Onset Symptoms	Management	Medication at Discharge
Campanile et al. [4]	2019	63 F	STEMI * and recent upper left limb embolism	Surgical thrombectomy and ascending aorta repair with autologous pericardial patch	ns *
Wang et al. [5]	2019	56 M	Asymptomatic, multiple prior asymptomatic cerebral infarctions	Surgical thrombectomy and ascending aorta replacement	ns
Asahara et al. [6]	2019	63 M	Transient left hemiplegia and disturbance of consciousness due to acute ischemic stroke and right renal infarction	Surgical thrombectomy and ascending aorta replacement	ns
Yang et al. [7]	2020	49 M	Chest discomfort for 5 days	Surgical thrombectomy and ascending aorta and proximal arch replacement	None
Frisoli et al. [8]	2020	48 M	Dysarthria and persistent right upper and lower extremity weakness due to left MCA * embolism	Percutaneous aspiration thrombectomy	Rivaroxaban
Ivanov et al. [9]	2020	51 F	Ischemia of left lower limb and subsequent superior mesenteric artery embolism	Surgical thrombectomy	ns
Furuta et al. [10]	2020	65 M	Stroke in the bilateral cerebral hemisphere and right cerebellar hemisphere	Antithrombotic	Oral anticoagulation ns
Kawai et al. [11]	2020	65 M	Dizziness, dysarthria, and disability of left hand due to cerebral infarction	Surgical thrombectomy and ascending aorta replacement	ns
Gueldich et al. [12]	2020	43 F	Left upper limb recurrent ischemia	Surgical thrombectomy and ascending aorta repair with Dacron patch	ns
	2020	63 F	Left upper limb ischemia, splenic infarction, and right renal artery embolism	Surgical thrombectomy and ascending aorta replacement	ns
Issa et al. [13]	2021	83 F	STEMI (LAD *) and stoke	Anticoagulant	ASA and warfarin
Koutroulou et al. [14]	2021	51 F	Transient episode of aphasia and subsequent stroke due to left MCA embolism	Intravenous thrombolysis (IVT) with alteplase	Warfarin (40 days) and after ASA
Quach et al. [15]	2021	53 M	Upper abdominal pain and intermittent chest pain	Surgical thrombectomy	Rivaroxaban
Dai et al. [16]	2021	59 M	Right lower limb embolism	Surgical thrombectomy and ascending aorta replacement	Rivaroxaban (3 months)
Oki et al. [17]	2021	65 M	Nausea, vomiting, and dizziness due to cerebral embolism	Surgical thrombectomy (trap door aortic incision)	Warfarin
Karkos et al. [18]	2021	50 M	Right hemispheric stroke	Anticoagulant	ns
Christou et al. [19]	2021	50 M	Weakness of left upper limb, palsy of left facial nerve, and left homonymous hemianopsia due to multiple cerebral ischemic strokes	Anticoagulant	Warfarin
Prasad et al. [20]	2021	60 F	Stroke due to left MCA embolism	Anticoagulation	ASA and clopidogrel
Abe et al. [21]	2021	62 M	Asymptomatic, admitted for hypoglycemic attack	Surgical thrombectomy and ascending aorta replacement	Warfarin (3 months)
Shoda et al. [22]	2021	54 M	STEMI (LAD) and multiple acute cerebral infarctions in both hemispheres and left MCA embolism	Surgical thrombectomy and ascending aorta replacement	ASA and warfarin
Moldovan et al. [23]	2021	50 M	Inferior STEMI (RCA *), vision impairment and restlessness	Surgical thrombectomy and CABG *	Warfarin (1 month), ASA and Clopidogrel (1 year)
Takafuji et al. [24]	2021	90 F	NSTEMI * (LMCA * embolism) with chest pain and syncope	Drug-eluting stent and anticoagulation	Warfarin, ASA and Clopidogrel
Noda et al. [25]	2021	58 M	Deterioration of consciousness, right paresis, and global aphasia due to left MCA embolism	Anticoagulation	Warfarin
Hirata et al. [26]	2022	50 M	Splenic and left renal infarction, superior mesenteric artery occlusion, and subsequent acute cerebral infarction	Surgical thrombectomy	Warfarin
Bojko et al. [2]	2022	39 M	Stroke due to left PICA embolism	Anticoagulant and subsequent surgical thrombectomy and ascending aorta replacement	Warfarin
Neves et al. [27]	2022	48 M	Right upper limb ischemia	Surgical thrombectomy and ascending aorta replacement	Oral anticoagulation ns
Rathnayake et al. [28]	2022	45 F	Left upper limb ischemia and chest pain	Surgical thrombectomy	Warfarin
Ewers et al. [29]	2022	36 M	Chest pain and shortness of breath with findings of pulmonary embolism	Anticoagulation	Dabigatran
Akcelik et al. [30]	2023	47 M	Stroke due to right MCA embolism	Surgical thrombectomy and ascending aorta replacement	ASA and warfarin
	2023	60 M	Bilateral lower limb embolism and kidney embolism	Surgical thrombectomy	ASA and Rivaroxaban
	2023	61 F	Bilateral lower limb embolism	Surgical thrombectomy	Warfarin
	2023	36 F	NSTEMI (LAD)	Surgical thrombectomy and CABG	ASA and warfarin
Wakami et al. [31]	2023	44 F	Dysarthria due to multiple cerebral emboli	Surgical thrombectomy	ns
Sattar et al. [32]	2023	43 M	Shortness of breath and renal artery embolism	Anticoagulant	DOAC *
Thurston et al. [33]	2023	38 M	Severe central chest pain and slurred speech due to multiple cerebral infarcts	Anticoagulant	Apixaban
Li et al. [34]	2023	43 M	Slurred speech and paralysis of the right limb due to left cerebral stroke with scattered hemorrhagic lesions	Surgical thrombectomy and ascending aorta (AA) repair with bovine pericardial patch	Long-term oral anticoagulants
Xiong et al. [35]	2023	53 M	Numbness and pain in right upper limb	Surgical thrombectomy (longitudinal aortic incision) and ascending aorta replacement	ns
Barbarossa et al. [36]	2024	49 M	STEMI (LAD) and bilateral pulmonary embolism	Surgical thrombectomy	ns
Johno et al. [37]	2024	64 M	Left hemiparesis due to right MCA embolism	Mechanical thrombectomy, surgical thrombectomy, and ascending aorta replacement	ASA and warfarin
Egbe et al. [38]	2024	50 M	Acute mesenteric ischemia	Anticoagulant	Apixaban
Liu et al. [39]	2024	58 M	Excruciating chest pain	Dual antiplatelet and anticoagulant	ASA and warfarin
Inoue et al. [40]	2024	49 M	Left hemiplegia, left facial palsy, dysarthria, and left hemispatial neglect due to right MCA embolism	Antithrombotic	ns

* MCA, middle cerebral artery; STEMI/NSTEMI, ST-elevation myocardial infarction/non-ST-elevation myocardial infarction; LMCA, left main coronary artery; LAD, left anterior descending; RCA, right coronary artery; CABG, coronary artery bypass graft; DOAC, direct oral anticoagulants; ns, not specified; M, male; F, female.

**Table 2:** Comparison of medical and surgical treatment.

Medical Treatment	Surgical Treatment
**Advantages:** – Allows the treatment of elderly patients with comorbidities and prohibitive operative risk – Great effectiveness on acute thrombi – More effective on descending thoracic aortic thrombi	**Advantages:** – Higher rates of pathology eradication – More effective on pedunculated and mobile thrombi – Various approaches thanks to the application of new technologies
**Disadvantages:** – Higher pathology recurrence and persistence, and further need for surgical treatment – Ineffective on chronic thrombi	**Disadvantages:** – Neurological complications and mortality due to the surgical procedure – Risk of perioperative embolization

## Data Availability

No new data were created or analyzed in this study.

## Abbreviations

The following abbreviations are used in this manuscript:

AAT	Ascending Aortic Thrombus
AD	Aortic Dissection
TAI	Traumatic Aortic Injury
CTA	Computed Tomography Angiography
